# Inhibition of the 60S ribosome biogenesis GTPase LSG1 causes endoplasmic reticular disruption and cellular senescence

**DOI:** 10.1111/acel.12981

**Published:** 2019-05-31

**Authors:** Asimina Pantazi, Andrea Quintanilla, Priya Hari, Nuria Tarrats, Eleftheria Parasyraki, Flora L. Dix, Jaiyogesh Patel, Tamir Chandra, Juan Carlos Acosta, Andrew J. Finch

**Affiliations:** ^1^ Cancer Research UK Edinburgh Centre, Institute of Genetics and Molecular Medicine University of Edinburgh Edinburgh UK; ^2^ MRC Human Genetics Unit, Institute of Genetics and Molecular Medicine University of Edinburgh Edinburgh UK

## Abstract

Cellular senescence is triggered by diverse stimuli and is characterized by long‐term growth arrest and secretion of cytokines and chemokines (termed the SASP—senescence‐associated secretory phenotype). Senescence can be organismally beneficial as it can prevent the propagation of damaged or mutated clones and stimulate their clearance by immune cells. However, it has recently become clear that senescence also contributes to the pathophysiology of aging through the accumulation of damaged cells within tissues. Here, we describe that inhibition of the reaction catalysed by LSG1, a GTPase involved in the biogenesis of the 60S ribosomal subunit, leads to a robust induction of cellular senescence. Perhaps surprisingly, this was not due to ribosome depletion or translational insufficiency, but rather through perturbation of endoplasmic reticulum homeostasis and a dramatic upregulation of the cholesterol biosynthesis pathway. The underlying transcriptomic signature is shared with several other forms of senescence, and the cholesterol biosynthesis genes contribute to the cell cycle arrest in oncogene‐induced senescence. Furthermore, targeting of LSG1 resulted in amplification of the cholesterol/ER signature and restoration of a robust cellular senescence response in transformed cells, suggesting potential therapeutic uses of LSG1 inhibition.

## 1 INTRODUCTION

Mammalian ribosomes are nucleoprotein complexes comprised of a large (60S) subunit and a small (40S) subunit that carry out the fundamental process of translation. The mature ribosome contains four ribosomal RNAs (rRNAs) and almost 80 proteins, and the complex process of ribosome biogenesis involves over 200 trans‐acting factors (reviewed in Kressler, Hurt, and Baßler (2017)). Transcription of rRNA precursors from tandem repeats of ribosomal DNA (rDNA) initiates ribosome biogenesis, and a complex sequence of events including sequential splicing and recruitment of rRNA‐associated proteins ensues. Mutations in genes that encode core ribosomal proteins or factors involved in ribosome biogenesis give rise to diseases that are collectively termed ribosomopathies. Examples of these inherited disorders include Treacher Collins syndrome, Diamond–Blackfan anaemia and Shwachman–Diamond syndrome (reviewed in Danilova and Gazda (2015)). The acquired myelodysplastic syndrome 5q‐, characterized by a deletion of a region of chromosome 5q, is also considered a ribosomopathy due to the presence of the *RPS14* gene in the deleted region and the phenotypic recapitulation of much of the disease phenotype upon deletion of *RPS14* alone (Barlow et al., 2010; Ebert et al., 2008). Given the requirement for ribosome biogenesis in cellular growth and proliferation, the causative mutation in these diseases is clearly detrimental to the cell. However, the pathology that arises in these ribosomopathies is, in many cases, caused by activation of the p53 pathway in response to the primary lesions (Barkic et al., 2009; Barlow et al., 2010; Jones et al., 2008). The exact nature of the stresses that activate the p53 pathway in the ribosomopathies remains undefined.

Regulation of ribosome biogenesis occurs primarily at the level of the transcriptional complexes that are recruited to the rDNA. The majority of rRNA is produced by RNA polymerase I‐mediated transcription, and this activity requires recruitment of TIF‐1A (transcription initiation factor 1A), UBF (upstream binding factor) and SL1 (selectivity factor 1) to rDNA promoter regions. All of these factors are regulated by phosphorylation, and they thereby integrate signals from the MAP kinase and mTOR pathways (Hannan et al., 2003; Mayer, Zhao, Yuan, & Grummt, 2004; Zhao, Yuan, Frödin, & Grummt, 2003). In addition, UBF is activated through interaction with cMyc (Poortinga et al., 2004) and inhibited by the Rb (Cavanaugh et al., 1995; Voit, Schäfer, & Grummt, 1997) and p53 (Budde & Grummt, 1999; Zhai & Comai, 2000) pathways. Accordingly, deregulation of ribosome biogenesis is commonly seen in cancer and the histochemical AgNOR test (for silver‐binding ArGyrophilic Nucleolar Organiser Regions) is used for staging and prognosis in many cases (Pich, Chiusa, & Margaria, 2000). The increased ribosome biogenesis observed in cancer has encouraged the idea that inhibition of this process could represent a therapeutic strategy in cancer therapy. Indeed, a small‐molecule inhibitor of RNA polymerase I, CX‐5461, has shown promise in this regard (Bywater et al., 2012; Drygin et al., 2011).

**Figure 1:**
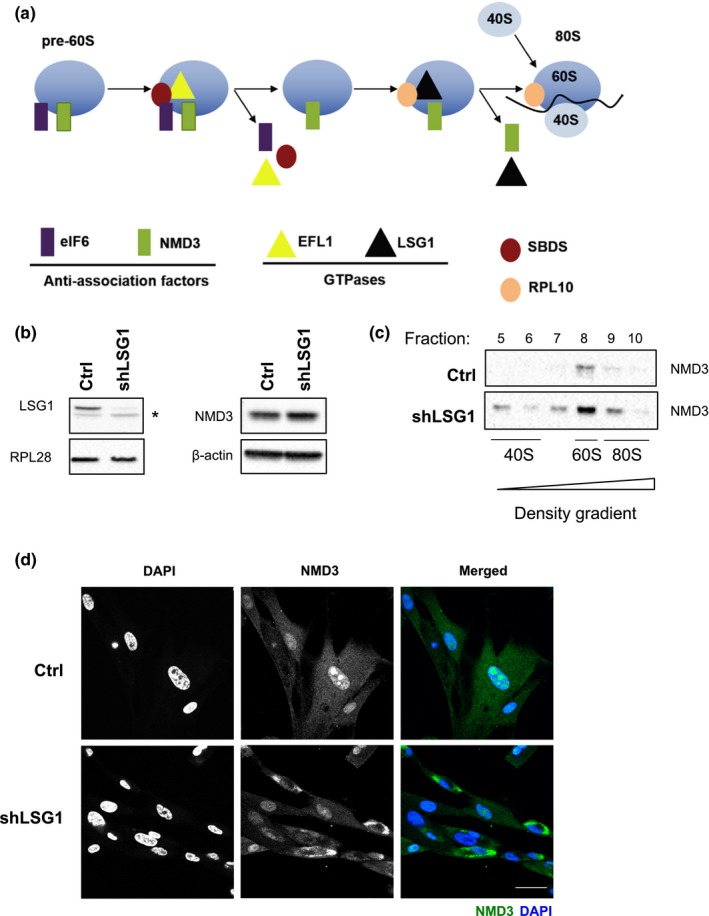
Knockdown of LSG1 inhibits NMD3 release from the ribosomal 60S subunit. (a) Schematic of the late cytoplasmic reactions of 60S subunit maturation. The cytoplasmic pre‐60S subunit carries the anti‐association factors eIF6 and NMD3. Recruitment of the factor SBDS and the GTPase EFL1 leads to eviction of eIF6 in a reaction catalysed by hydrolysis of GTP. SBDS stimulates GTP hydrolysis by EFL1, which induces a rotation in the structure of SBDS, resulting in conformational changes and eIF6 release. RPL10 and the GTPase LSG1 then bind to the subunit leading to eviction of NMD3, again catalysed by GTP hydrolysis. RPL10 is retained on the 60S subunit, and the mature 80S ribosome is formed. (Adapted from Finch et al., 2011 and Hedges et al., 2005) (b) Western blot analysis shows the knockdown of LSG1 in HEK 293 cells. The asterisk denotes a nonspecific band. RPL28 was used as a reference protein. A blot for NMD3 in IMR90 cells with β‐actin as control is also shown. (c) Western blot analysis shows the levels of NMD3 and RPS14 across sequential fractions (5–10) collected from sucrose gradients in control and shLSG1 conditions. Extracts were normalized by spectrophotometry at 254 nm prior to loading. The NMD3 in fraction 8 corresponds to the localization of 60S monomers. (d) Immunostaining for NMD3 in MRC5 cells (control and shLSG1), followed by confocal microscopy, reveals relocalization of NMD3 to the cytoplasm following LSG1 knockdown. Scale bar: 50 μm

We identified the GTPases involved in the cytoplasmic maturation of the 60S ribosomal subunit as plausible targets for therapeutic intervention. These GTPases catalyse the release of two anti‐association factors that are loaded onto the 60S particle in the nucleus and that are removed in the cytosol at the last stages of 60S maturation (Finch et al., 2011; Lo et al., 2010; Figure 1a). EFL1 leads to eviction of the anti‐association factor eIF6 from the immature pre‐60S in a reaction that requires the SBDS cofactor and GTP hydrolysis (Finch et al., 2011), whilst LSG1 catalyses the eviction of the anti‐association factor NMD3 in a reaction requiring RPL10, which stays associated with the ribosome (Hedges, West, & Johnson, 2005; Ma et al., 2017; Malyutin, Musalgaonkar, Patchett, Frank, & Johnson, 2017). Following removal of eIF6 and NMD3, the mature 60S subunit can then join the translating pool of ribosomes and both anti‐association factors are returned to the nucleus to participate in subsequent rounds of 60S biogenesis.

Here, we report that knockdown of LSG1 and other components of the 60S maturation pathway promotes a robust activation of cellular senescence. This senescence response is characterized by activation of the p53 and p16/Rb pathways and by a highly restricted SASP lacking the NF‐κB‐driven pro‐inflammatory cytokines and chemokines. shLSG1 also promotes a striking upregulation of the cholesterol biosynthesis pathway and genes involved in endoplasmic reticulum (ER) organization, and this is accompanied by a disruption of the reticular morphology of the ER. Indeed, RPL10 and LSG1 have been shown to associate with ribosomes at the rough ER (Loftus, Nguyen, & Stanbridge, 1997; Reynaud et al., 2005) and our data suggest that loss of LSG1 significantly impacts upon ER homeostasis. Finally, we provide evidence that inhibition of 60S maturation can restore a senescence response in oncogene‐transformed cells that have already bypassed oncogene‐induced senescence (OIS).

## 2 RESULTS

### 2.1 Knockdown of LSG1 inhibits NMD3 release from the ribosomal 60S subunit

The enzymes that catalyse the final cytoplasmic reactions in the maturation of the large (60S) ribosomal subunit (Figure 1a) represent possible targets for therapeutic inhibition. Accordingly, we chose RNAi rather than gene deletion as a strategy to mimic pharmacological inhibition because it can be efficient, yet not absolute. Since LSG1 catalyses the release of NMD3 from the cytoplasmic pre‐60S particle, knockdown of LSG1 should result in failure to release NMD3 and therefore to its cytosolic sequestration (Hedges et al., 2005; Ma et al., 2017; Malyutin et al., 2017). Infection of cells with a lentiviral vector encoding a shRNA to LSG1 led to efficient knockdown of the protein, as assessed by Western blot (Figure 1b). Knockdown of LSG1 resulted in minimal upregulation of NMD3 (Figure 1b), but rather to an increase in association of NMD3 with the 60S subunit fraction as assessed by sucrose density gradient separation of ribosomal subunits (Figure 1c). NMD3 is loaded onto pre‐60S subunits in the nucleus (Gadal et al., 2001; Ho, Kallstrom, & Johnson, 2000), and immunofluorescent staining of control cells showed a nuclear/nucleolar staining pattern for NMD3 (Figure 1d). Knockdown of LSG1 led to relocalization of NMD3 to the cytoplasm (Figure 1d), consistent with its retention on maturing cytoplasmic pre‐60S particles due to loss of LSG1‐mediated release. This relocalization of Nmd3 from nucleus to cytoplasm is also observed in yeast lacking Lsg1 (Hedges et al., 2005) and is diagnostic of the defect in this maturation reaction.

### 2.2 Impairment of 60S maturation triggers a robust cellular senescence response

**Figure 2:**
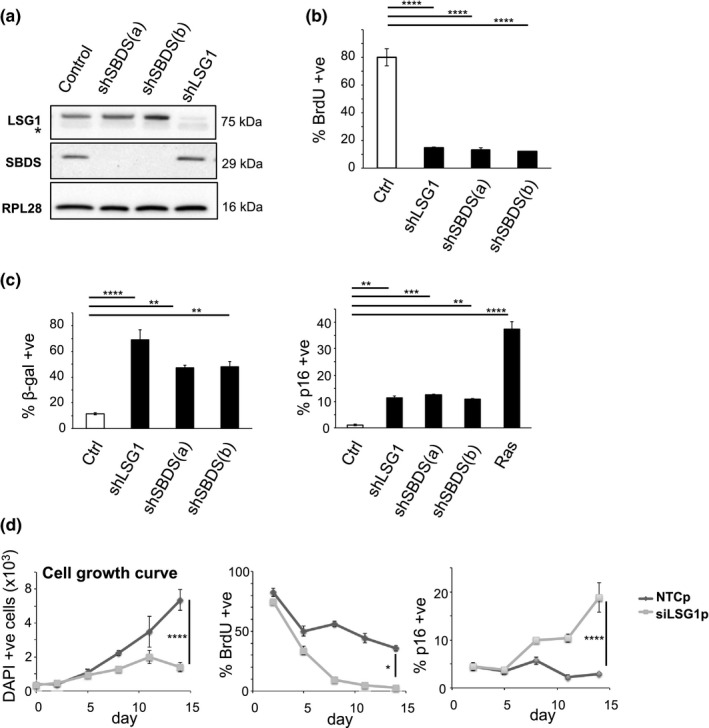
Knockdown of LSG1 and SBDS induces senescence. (a) Western blot showing the efficiency of LSG1 and SBDS knockdown in MRC5 cells induced by the hairpins shLSG1, shSBDS(a) and shSBDS(b). The asterisk denotes a nonspecific band in the LSG1 blot. RPL28 was used as a reference protein. (b) High content imaging analysis of BrdU incorporation and immunostaining in MRC5 cells with LSG1 and SBDS downregulation, 7 days postinfection. The cells were treated with 50 mM BrdU for 16 hr. (c) The senescence‐associated β‐galactosidase assay was performed 7 days postinfection. Images were taken using phase contrast microscopy, and the number of cells that were positive for the blue precipitate was counted. The bar chart on the right shows high content imaging analysis of p16 immunostaining. Ras‐transduced cells were used as a positive control for p16 induction. (d) Time course experiment (time points: d0, d2, d5, d8, d11, d14) using a siRNA SMARTpool for LSG1 (siLSG1p). Cell growth (DAPI stain), BrdU incorporation and p16 expression were monitored throughout the time course using high content microscopy. Error bars show standard deviation of 3 biological replicates. Statistical significance was calculated using one‐way ANOVA with Dunnett's (Figure 2b,c) or Sidak's (Figure 2d) multiple comparisons tests. *p < 0.05, **p < 0.01, ***p < 0.001, and ****p < 0.0001

We generated additional shRNAs to SBDS (the cofactor for EFL1 (Finch et al., 2011)) to target 60S maturation and assessed their knockdown by Western blot: we obtained two shRNAs that were efficient for SBDS (Figure 2a). We introduced the shRNAs into primary human MRC5 fibroblasts through lentiviral transduction to impair 60S maturation and assessed their growth. Several days after viral infection, we noticed that impairment of 60S maturation led to a sparse culture and spreading of the cells with morphology that resembled cellular senescence. Analysis of BrdU incorporation using high content microscopy revealed that knockdown of LSG1 and SBDS led to a cell cycle arrest (Figures 2b and S1a) and this was accompanied by activation of acidic β‐galactosidase activity and accumulation of p16 mRNA and protein (Figures 2c, S1a,b and S2a), indicating a senescence response. In addition, we also observed increased staining for p53, p21 and the DNA damage response marker pST/Q (Figures S1a and S2b). Furthermore, although the shSBDS(b) shRNA gave a less robust response with p53 and p21 immunofluorescence, p21 mRNA was induced consistent with activation of the p53 pathway (Figure S2a,b). Although activation of the p53 pathway can lead to apoptosis under certain circumstances, we observed no induction of apoptosis by knockdown of LSG1 (Figure S2c). Senescence is characterized by ongoing, rather than transient, growth arrest, and we confirmed the continuous nature of the shLSG1‐induced growth defect through assessment of BrdU incorporation and p16, p53 and p21 immunoreactivity in a time course over 15 days (Figures 2d and S2d). To confirm the specificity for LSG1 in this process, we first generated and utilized a second shRNA to LSG1 (Figure S3a) and again observed induction of acidic β‐galactosidase activity (Figure S3b) and reduction in BrdU staining (Figure S3c). Next, we used siRNA SMARTpools to EFL1 and LSG1 (Figure S4a) and observed the expected reduction in BrdU incorporation (Figure S4b), induction of acidic β‐galactosidase activity (Figure S4c) and induction of p16 immunoreactivity (Figure S4d). Deconvolution of the LSG1 siRNA pools revealed two independent siRNAs that knocked down LSG1 (Figure S5a), reduced BrdU incorporation, and induced p16 and p53 (Figure S5b). Taken together, these results demonstrate that inhibition of 60S ribosomal subunit maturation triggers a robust cellular senescence response.

### 2.3 The senescence response to inhibition of 60S maturation is p53‐dependent in primary cells

**Figure 3:**
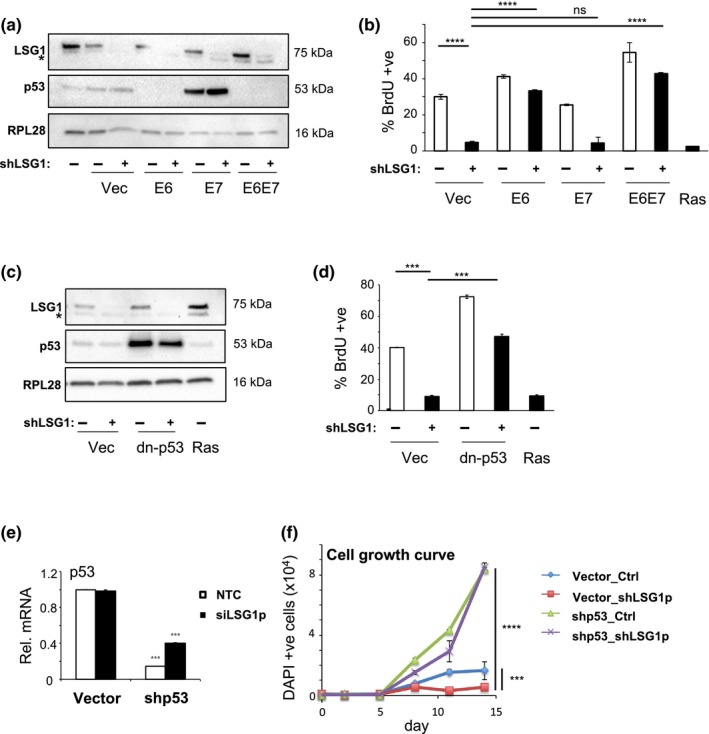
The senescence response induced by LSG1 knockdown is p53‐dependent. (a) Western blots for LSG1, p53 and RPL28 in MRC5 cells transduced with shLSG1 and/or HPV E6, E7 or E6E7 (the asterisk denotes a nonspecific band). (b) BrdU incorporation was measured by high content imaging in cells transduced as in (a) above. K‐RAS^G12V^‐transduced cells were used as a positive control for growth arrest. (c) Western blots for LSG1, p53 and RPL28 in MRC5 cells transduced with shLSG1 and/or a dominant‐negative p53 construct (dn‐p53) (the asterisk denotes a nonspecific band). Ras retroviral overexpression is included as a positive control. (d) BrdU incorporation was measured by high content imaging in cells transduced as in (c) above. (e) qRT–PCR analysis of MRC5 cells transduced with shLSG1 and/or shp53 for the quantification of p53 transcript levels. (f) Time course experiment for the study of the growth levels of the above (e) cells, using high content imaging to measure DAPI stain. Time points: d0, d2, d5, d8, d11, d14. Error bars show standard deviation of 3 biological replicates. Statistical significance was calculated using two‐tailed *t* tests or one‐way ANOVA with Dunnett's (Figure 3b) or Sidak's (Figure 3f) multiple comparisons tests. *p < 0.05, **p < 0.01, ***p < 0.001, and ****p < 0.0001

The two main pathways that implement most aspects of replicative and oncogene‐induced senescence responses are the p16/retinoblastoma (RB) and p53 pathways (Salama, Sadaie, Hoare, & Narita, 2014). As described above, we observed that both pathways were activated by knockdown of LSG1 and we set out to determine which of these pathways was required for induction of senescence under this condition. The viral oncoproteins E6 and E7 from the human papillomavirus inhibit the p53 and Rb pathways, respectively, and are well‐established tools for the determination of function of these pathways (Boulet, Horvath, Vanden, Sahebali, & Bogers, 2007). We infected primary human fibroblasts with retroviral vectors expressing E6, E7 or an E6‐E7 fusion protein (Acosta et al., 2008) and then with lentiviral shRNA to LSG1. Both viral constructs were functional since expression of E6 abrogated p53 expression, whilst E7 expression enhanced p53 levels as previously described (Demers, Halbert, & Galloway, 1994; Figure 3a). Loss of p53 function leads to bypass of replicative and oncogene‐induced senescence (Bond, Wyllie, & Wynford‐Thomas, 1994; Serrano, Lin, McCurrach, Beach, & Lowe, 1997), and similarly, E6 expression led to maintained BrdU incorporation upon LSG1 knockdown (Figure 3b), indicating p53 dependence. Expression of E7, on the other hand, did not rescue the inhibition of cell cycle elicited by LSG1 knockdown. We confirmed the p53 dependence of the senescence response using a C‐terminal, dominant‐negative fragment of p53, which leads to stabilization of the endogenous p53 protein through inhibition of its function (Figure 3c). Once again, inhibition of the p53 pathway led to bypass of shLSG1‐induced proliferative arrest (Figure 3d). Finally, we used shRNA to p53 to follow the growth characteristics of cell lines transduced with shRNA to LSG1 (Figure 3e). p53 knockdown resulted in greatly accelerated growth rates in vector control cells, and the knockdown of LSG1 failed to inhibit growth in these cells (Figure 3f).

### 2.4 Knockdown of LSG1 induces a senescent transcriptional response

**Figure 4:**
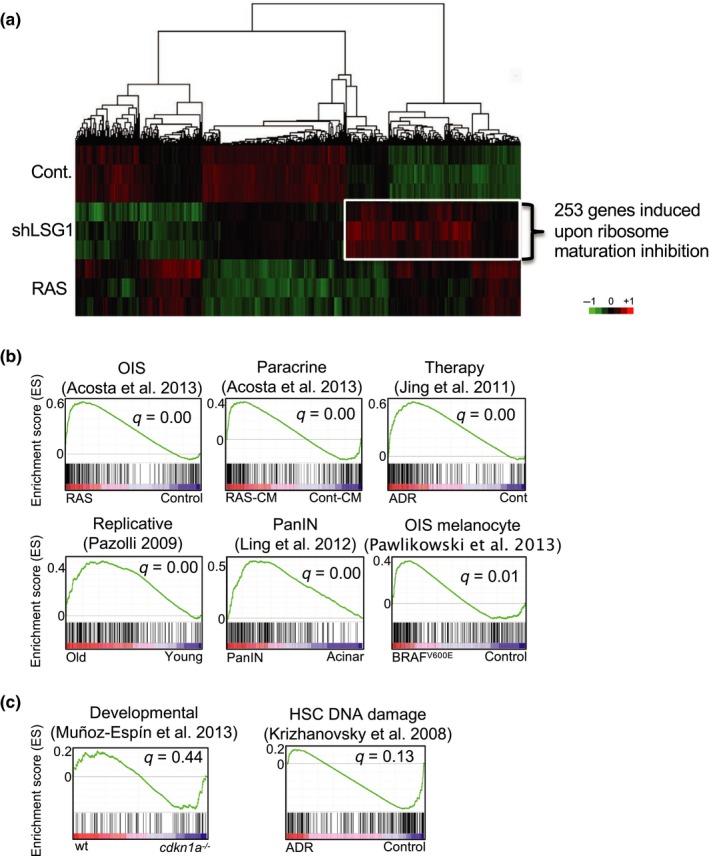
A signature of genes induced by LSG1 knockdown is common with other senescence responses: (a) Hierarchical clustering of mRNA profiles from cells transduced with K‐RAS^G12V^, shLSG1 and vector control (Cont.) in MRC5 cells showing genes changing significantly (Adj.*p* < 0.01) between shLSG1 and control (GSE128055). A signature of 253 genes induced by shLSG1 is highlighted. Data represent 3 experimental replicates. (b) GSEA plots showing that a signature of 253 genes derived from MRC5 cells undergoing shLSG1‐induced senescence (described in a.) is significantly enriched in multiple forms of senescence. (*q* represents false discovery rate (FDR)). (c) GSEA plots showing that a signature of 253 genes derived from MRC5 cells undergoing shLSG1‐induced senescence (described in a.) is not significantly enriched during developmental senescence or DNA damage‐induced senescence. (*q* represents false discovery rate (FDR))

The transcriptional responses to several triggers of senescence have recently been reported (Acosta et al., 2013, 2008; Hoare et al., 2016; Muñoz‐Espín et al., 2013). Therefore, in order to gain mechanistic insight into the molecular cause of the senescence elicited by inhibition of 60S maturation, we performed transcriptomic analysis of shLSG1 cells. In particular, we sought to investigate molecular signatures that were shared with other forms of senescence. Senescence was induced in primary human MRC5 fibroblasts through transduction of shLSG1 and through overexpression of K‐Ras^G12V^ as a positive control for oncogene‐induced senescence. Extraction of RNA was followed by AmpliSeq library preparation and IonTorrent sequencing of amplicons, and the differential expression of cells expressing shLSG1, oncogenic K‐Ras^G12V^ and control was analysed (Figure 4a and Data S1). Global gene expression clustering revealed clear differences between the two senescent states (Figure 4a), and we therefore assessed enrichment of the signature of 253 genes induced by LSG1 knockdown (Figure 4a and Data S2) in the transcriptomes of previously reported triggers of senescence. We interrogated preranked gene expression lists from several systems in which senescence was induced, including OIS (Acosta et al., 2013; Pawlikowski et al., 2013), replicative senescence (Pazolli et al., 2009), paracrine senescence (Acosta et al., 2013), drug‐induced senescence (Jing et al., 2011) and pancreatic intraepithelial neoplasia (Ling et al., 2012). The preranked lists of genes were used to perform gene set enrichment analysis (GSEA) of the 253 genes induced by shLSG1 and in almost all cases showed enrichment with a false discovery rate‐adjusted (FDR) *Q*‐value of 0.01 or below (Figure 4b). The two exceptions that did not show statistically significant enrichment were developmental senescence (Muñoz‐Espín et al., 2013) and DNA damage‐induced senescence in hepatic stellate cells (Krizhanovsky et al., 2008; Figure 4c). Thus, the transcriptional response to LSG1 knockdown contains a strong senescent signature that is shared with multiple forms of senescence that arise in vitro and in vivo.

### 2.5 Knockdown of LSG1 induces production of a restricted SASP

**Figure 5:**
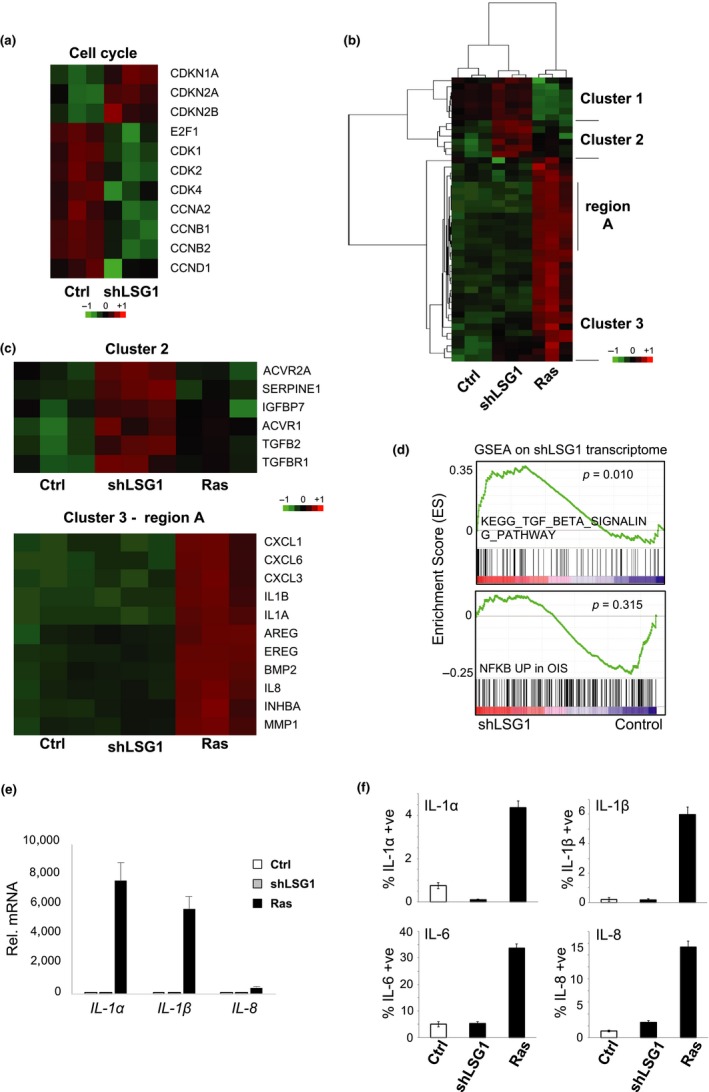
Transcriptomic analysis reveals a robust senescent transcriptional response with a restricted SASP upon LSG1 knockdown. (a) Regulation of antiproliferative and proliferative cell cycle‐related transcripts by shLSG1 in MRC5 cells. (b) Clustering of transcript levels of SASP factors. (c) Cluster 2 contains a set of mRNAs that are specific for shLSG1 (vs. K‐RAS^G12V^) that includes TGFB2 and related genes. Cluster 3, region A is OIS‐specific and is comprised of NF‐κB‐driven canonical SASP genes. (d) GSEA of the transcriptome of MRC5 cells transduced with shLSG1 compared to control showing significant enrichment for the TGFB signalling pathway (KEGG pathway) and no significant enrichment for the OIS‐associated NF‐κB signature (Chien et al., 2011) (e) qPCR analysis of the above cells for the quantitation of IL‐1α, IL‐1β and IL‐8 transcript levels. (f) High content imaging analysis of the SASP factors IL‐1α, IL‐1β, IL‐6 and IL‐8. KRAS^G12V^ retroviral overexpression was included as a positive control. Error bars show standard deviation of 3 biological replicates

As expected, we observed a marked antiproliferative signature characterized by upregulation of CDK inhibitors and downregulation of *E2F1*, cyclins and cyclin‐dependent kinases (Figure 5a). One of the hallmarks of senescent cells is the release of a cocktail of pro‐inflammatory cytokines and chemokines, collectively termed the SASP. We analysed our transcriptomic data in more detail for genes previously identified as SASP‐related or generally involved in inflammation (Acosta et al., 2013). This analysis revealed a lack of most of the canonical SASP factors involved in OIS and revealed three distinct gene clusters (Figure 5b)—including one that was upregulated upon LSG1 knockdown but only weakly (or not at all) with OIS (Figure 5c, cluster 2) and one specific for OIS (Figure 5c, cluster 3). The shLSG1‐specific cluster (cluster 2) included *TGFβ2* and *TGFβR1* as well as the other TGFβ family receptors *ACVR1* and *ACVR2a* and the TGFβ target genes *SERPINE1* and *IGFBP7* (Figure 5c). This was supported by gene set enrichment analysis, which indicated significant enrichment of genes associated with the TGFβ signalling pathway (Figure 5d) and qRT–PCR analyses that verified upregulation of *SERPINE1*, *TGFB2* and *IGFBP7* (Figure S6a). The OIS‐specific transcriptome cluster included strongly pro‐inflammatory cytokines and chemokines (Figure 5c, cluster 3, region A), indicative of the strong NF‐κB‐driven SASP program in OIS. Gene set enrichment analysis between the shLSG1 transcriptome and the OIS NF‐κB programme showed no significant induction of these genes upon knockdown of LSG1 (Figure 5d), and this lack of key NF‐κB‐driven SASP components was confirmed at the mRNA (Figure 5e) and protein (Figure 5f) levels. Thus, impairment of 60S maturation through LSG1 knockdown elicits a restricted SASP centred around TGFβ/activin signalling.

### 2.6 Increased translation coincident with senescence occurs in cells with knockdown of LSG1

**Figure 6:**
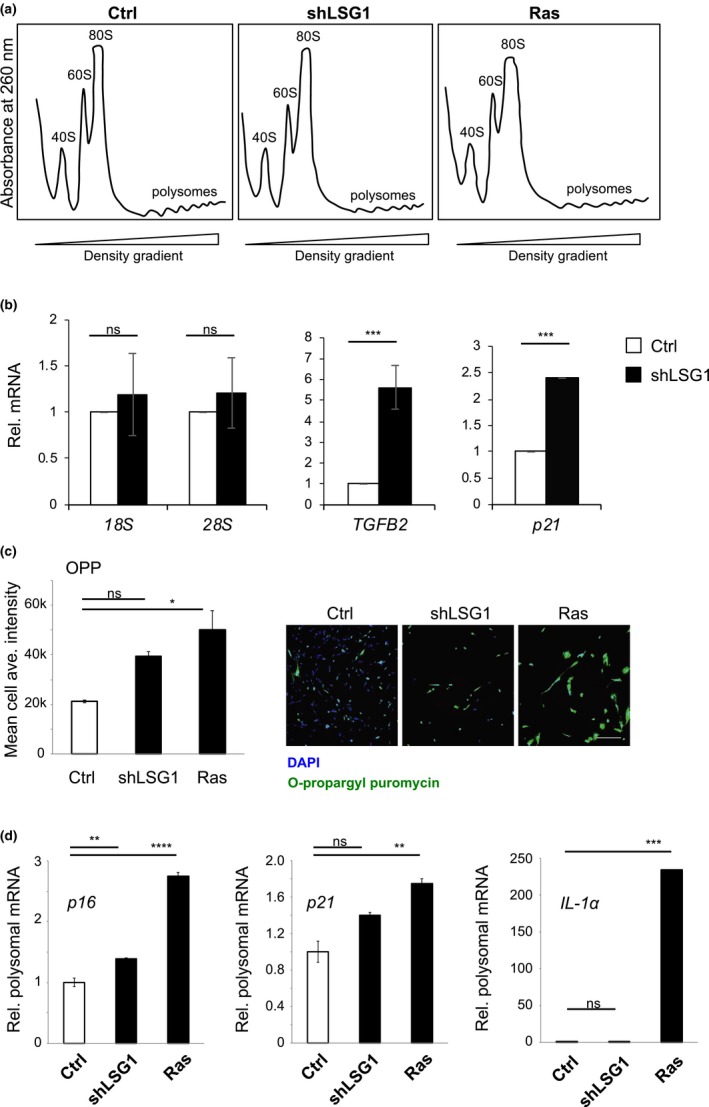
Knockdown of LSG1 does not inhibit global translation. (a) Polysome profiling of MRC5 cells at senescence triggered by shLSG1 or K‐RAS^G12V^ after 7 days. (b) qPCR analysis of the above cells for the quantitation of ribosomal 18S and 28S transcript levels to assess total ribosomal subunit composition, alongside TGFB2 and p21 confirming the senescence response. (c) Analysis of translational activity using O‐propargyl puromycin (OPP) and high content imaging. Quantitation of mean cell average intensity from images obtained. Representative images are provided. Scale bar: 200 μm. (d) qPCR analysis of polysome‐associated transcripts for the senescence markers p16, p21 and IL‐1α. Error bars show standard deviation of 3 biological replicates. Statistical significance was calculated using two‐tailed *t* tests or one‐way ANOVA with Dunnett's multiple comparisons tests (Figure 6c,d). *p < 0.05 **p < 0.01, ***p < 0.001, and ****p < 0.0001

Since LSG1 catalyses a key step in the maturation of the 60S ribosomal subunit, a possible mechanism for generation of stress leading to senescence could be a lack of 60S subunits and consequent translational insufficiency. We transduced cells with vector, shLSG1 or KRas^G12V^, awaited the onset of senescence and then performed polysome profiling to assess the ribosomal composition of the cells. We observed no qualitative differences between the conditions, except perhaps for a marginal reduction in peak height for the 60S subunit upon shLSG1 transduction (Figure 6a), suggesting that senescence occurred well before impairment of 60S maturation could affect overall polysomal composition. We also employed qRT–PCR for the 18S and 28S rRNAs to quantify differences in total ribosomal subunit composition, and once again, we found no significant change in either subunit upon knockdown of LSG1, despite confirmation of onset of senescence by induction of *TGFB2* and *p21* (Figure 6b). In order to assess the impact on translation, we used O‐propargyl puromycin (OPP) to label actively translating ribosomes and we quantified OPP incorporation by high content microscopy. Rather than causing a reduction, knockdown of LSG1 gave rise to an elevated translation rate (Figure 6c). Oncogene‐induced senescence induced by H‐Ras^G12V^ also led to increased translation, consistent with previous reports that senescence is a cellular state associated with high translational and metabolic activity (Dörr et al., 2013; Herranz et al., 2015; Laberge et al., 2015; Narita et al., 2011). We harvested the polysomal fractions from our profiling experiment and performed qRT–PCR to assess whether mRNAs involved in the senescence response were being actively translated. As expected, polysome‐associated mRNAs for p16 and p21 were elevated in both of the senescent conditions, whereas IL‐1α, the master regulator of the SASP (Laberge et al., 2015), was associated with polysomes in the OIS sample alone (Figure 6d). Thus, the senescence response to impairment of 60S ribosomal subunit maturation is not triggered by ribosome depletion or translational insufficiency.

### 2.7 60S inhibition leads to disruption of ER homeostasis and morphology

**Figure 7:**
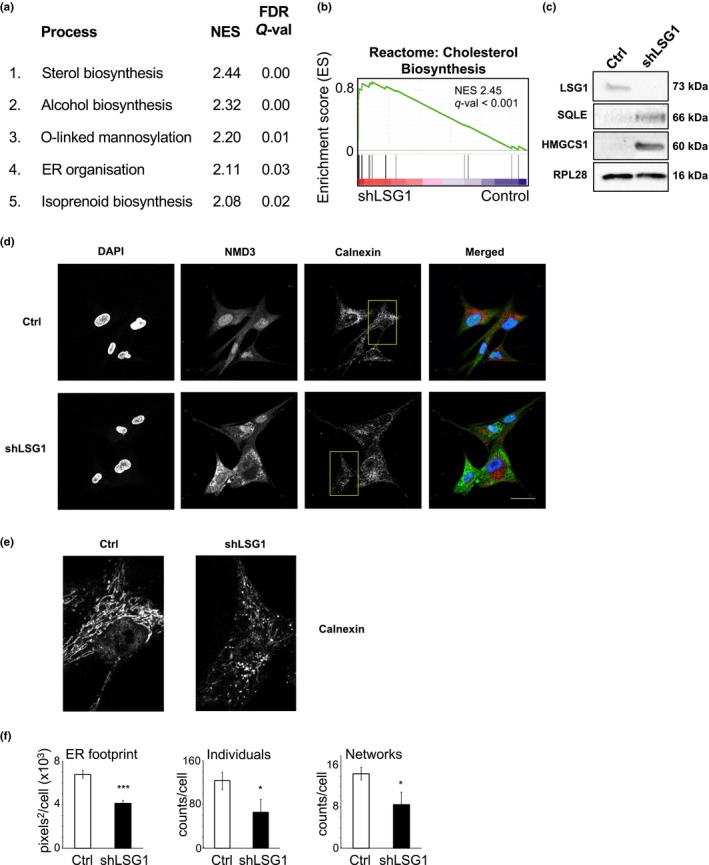
Knockdown of LSG1 leads to upregulation of cholesterol biosynthesis pathways and homeostatic alterations in the ER apparatus. (a) Gene set enrichment analysis (GSEA), ranked by normalized enrichment score (NES), revealed the top 5 upregulated biological processes as a result of LSG1 knockdown. The false discovery rate (FDR) yields the *Q*‐value for statistical significance. (b) GSEA diagram of the cholesterol biosynthesis signature upon LSG1 knockdown as described in (a). (c) Western blot for LSG1, RPL28 and the cholesterol biosynthesis enzymes SQLE and HMGCS1 in shLSG1‐transduced MRC5 cells. (d) Immunofluorescence staining for calnexin in MRC5 cells transduced with control and with shLSG1, imaged by confocal microscopy. Scale bar: 50 μm. (e) High magnification images of the regions indicated in (d) stained for calnexin. (f) FIJI‐based analysis of the ER skeleton in the cells above, using the MiNA plugin (Valente et al., 2017). Error bars denote *SEM* of three biological replicates. Statistical significance is calculated using two‐tailed *t* tests. *p < 0.05, **p < 0.01, and ***p < 0.001

In addition to the targeted analyses of transcriptomic data described above, we utilized global GSEA to shed light upon the cellular response to LSG1 knockdown. This analysis revealed a striking upregulation of processes that occur at the ER (top five processes shown in Figure 7a), in particular the cholesterol biosynthesis pathway (Figure 7b). Indeed, 7 of the top 25 upregulated genes in our analysis encoded members of the cholesterol synthesis pathway and almost every member of the pathway was upregulated (Figure S6b). We also confirmed upregulation of squalene epoxidase (SQLE) and hydroxymethylglutaryl‐CoA synthase (HMGCS1) protein (Figure 7c). We were unable to assess the contribution of the cholesterol biosynthesis pathway to the induction of senescence as dual knockdown of LSG1 and individual cholesterol biosynthesis genes was toxic to the cells. The striking enrichment of the cholesterol biosynthesis and other ER‐related pathways in shLSG1‐induced senescence led us to look more closely at the morphology of the ER. LSG1 has been reported to predominantly localize to the ER (Reynaud et al., 2005), and its reaction partner RPL10 (also known as QM protein) has been shown to interact with ER‐associated ribosomal particles (Loftus et al., 1997). Immunofluorescent staining for the ER marker calnexin revealed the expected reticular morphology of the ER in control cells, but in shLSG1 cells where NMD3 was cytoplasmic, the ER appeared highly fragmented and punctate (Figure 7d,e). We quantified this effect using the MiNA plugin for ImageJ (Valente, Maddalena, Robb, Moradi, & Stuart, 2017) which analyses reticularity of cellular features. Upon knockdown of LSG1, we observed a reduction in ER footprint, number of individual ER components and number of ER networks, indicating a marked disruption of ER morphology (Figure 7f). Thus, knockdown of LSG1 leads to disruption of ER homeostasis and morphology.

### 2.8 The cholesterol biosynthetic and ER transcriptomic programmes are common to the senescence induced by inhibition of 60S maturation and OIS

**Figure 8:**
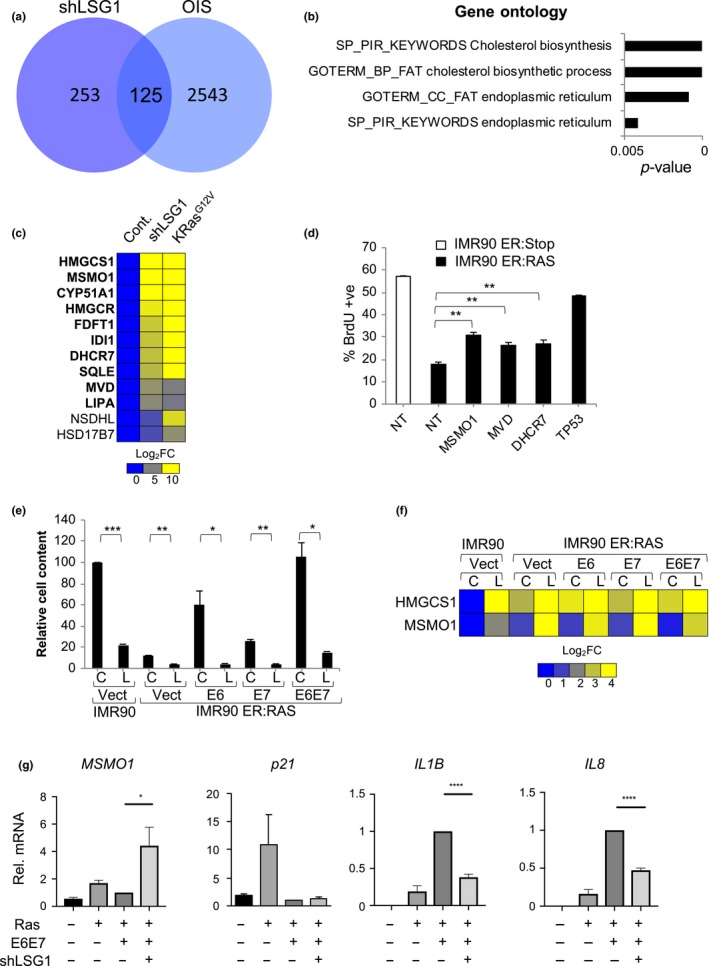
LSG1 targeting restores the cholesterol/ER senescent programme in H‐RAS^G12V^‐expressing cells that have bypassed senescence. (a) Venn diagram representing the number of genes commonly induced between shLSG1 knockdown induced senescence and OIS in MRC5 cells by AmpliSeq transcriptome analysis. (b) Bar graph representing the p‐value after functional annotation analysis of the most significant GO terms enriched in the 125 genes induced by shLSGI and oncogenic RAS in MRC5 cells as in a. Analysis was performed using the DAVID web resource. (c) Heat map representing mRNA fold change (Log_2_ scale) in the AmpliSeq expression profile of cholesterol biosynthesis genes in shLSG1‐ and RAS‐transduced MRC5 cells. Each sample represents the mean of 3 experimental replicates. Bold character genes represent significant changes in expression in both conditions. (d) BrdU proliferation assay of IMR90 ER:RAS or ER:Stop control cells 5 days after 4 hydroxytamoxifen (4OHT) treatment and siRNA SMARTpool transfection for the cholesterol biosynthesis genes MSMO1, MVD, DHCR7 and TP53 (as a positive control). Nontargeting (NT) siRNA SMARTpool was used as a negative control. Bars represent the mean of 3 experimental replicates. Error bars represent the *SEM*. (e) Proliferation assay showing relative cell content of cells transduced with shLSG1 (L) or control (C) lentiviral vectors in cells bypassing OIS. Bypass of OIS was achieved with retrovirus expressing HPV proteins E6, E7, E6E7 or neomycin control. Cells were seeded at low density, cultured for 14 days and stained with crystal violet (CV) as indicated. Bars represent the mean quantification of CV staining of three independent experiments. Error bars represent the *SEM*. (f) Heat map showing HMGCS1 and MSMO mRNA fold change (Log_10_ scale) by qRT–PCR from cells treated as in (e) above: C refers to control; L refers to shLSG1. (g) qRT–PCR analysis of IMR90 cells transduced with Vector control, Ras, Ras/E6E7 or Ras/E6E7/shLSG1. MSMO1, p21, IL1B and IL8 were measured. Statistical significance was calculated using two‐tailed *t* tests or one‐way ANOVA with Dunnett's multiple comparisons tests (Figure 8d). *p < 0.05, **p < 0.01, ***p < 0.001, and ****p < 0.0001

Oncogene‐induced senescence is driven by multiple cellular stress responses, including replication stress and DNA damage, metabolic and oxidative stresses (reviewed in Kuilman, Michaloglou, Mooi, and Peeper (2010)). We wished to ascertain whether we could detect signals of a stress response in our transcriptomic data that were conserved between shLSG1‐induced and oncogene‐induced senescence (OIS). We therefore compared the transcriptomes of cells that underwent senescence due to knockdown of LSG1 or OIS induced by K‐Ras^G12V^ to find genes that were upregulated in both cases. We found 125 genes upregulated in common between the two senescent programmes (Figure 8a), and we subjected these genes to gene ontology analysis. Strikingly, by far the most significant signature that emerged (Figure 8b) was cholesterol biosynthesis (*p*‐value = 1.13 × 10^−9^), followed by ER compartment (*p*‐value = 8.73 × 10^−4^). Since the gene sets for ER include most of the genes involved in cholesterol biosynthesis, the predominant shared component of the senescent transcriptomic response is an induction of cholesterol biosynthesis. We found that almost every gene in the cholesterol biosynthesis pathway was upregulated in both forms of senescence (Figure 8c), suggesting that the pathway may be of functional importance in the senescence response. We therefore undertook a restricted cholesterol biosynthetic siRNA screen for bypass of OIS, which we defined as an increase of 30% in BrdU incorporation compared to the senescent state. Several siRNAs from the pathway bypassed OIS (Figure S7a) and the three strongest candidates from the screen (*MSMO1*, *MVD* and *DHCR7*) showed robust and significant bypass of OIS (Figures 8d and S7b). Thus, activation of the cholesterol biosynthesis pathway is a tumour‐suppressive response that contributes to senescence induced by perturbation of 60S maturation and oncogenic Ras.

### 2.9 shLSG1 amplifies the cholesterol biosynthesis signature and induces senescence in cells that have bypassed OIS

A critical step in the transformation of cells expressing oncogenic Ras is the bypass of OIS through disruption of the p53 or RB pathways (Serrano et al., 1997). One (or both) of these canonical tumour‐suppressive pathways is inactivated in most cancers, and thus, for a prosenescent cancer therapy to be effective, it should be able to elicit tumour suppression independently of these two pathways. We wished to assess whether the induction of the cholesterol biosynthesis programme by inhibition of 60S maturation might provide such a tumour‐suppressive response. We therefore generated pretransformed cells through a combination of overexpression of H‐Ras^G12V^ and the human papillomavirus oncoproteins E6, E7 or an E6‐E7 fusion and then performed knockdown of LSG1. We observed that knockdown of LSG1 reduced cell content in the absence or presence of oncogenic Ras and that E6, E7 and E6‐E7 bypassed Ras‐induced growth arrest (Figures 8e and S7c), as expected. In the conditions where OIS was bypassed, shLSG1 elicited a marked growth arrest, even in the E6E7 line where both p53 and RB pathways are defective (Figures 8e and S7c). This growth arrest was accompanied by induction of the cholesterol biosynthesis pathway (shown for *HMGCS1* and *MSMO1* in Figure 8f), and acidic β‐galactosidase staining indicated that the reduced cell number was due to a senescence response (Figure S7d). More detailed analysis of the Ras/E6E7 cells revealed that although the cholesterol biosynthesis genes MSMO1, HMGCS1, SQLE and FDFT1 were already induced by Ras, shLSG1 led to their further induction (Figures 8g and S7e). p21 was not induced, due to p53 inactivation by E6, but notably, shLSG1 led to a reduction in levels of IL1B and IL8. Taken together, these data reveal that inhibition of 60S maturation elicits a p53‐independent senescence response in cells that have bypassed OIS and simultaneously restricts the potent pro‐inflammatory SASP driven by oncogenic Ras.

## 3 DISCUSSION

Here, we show that inhibition of 60S maturation leads to a robust induction of cellular senescence that is associated with perturbation of ER homeostasis and that this can elicit tumour suppression even in cells with bypass of OIS. The impairment of 60S ribosomal subunit maturation upon knockdown of LSG1 was verified by relocalization of NMD3 to the cytoplasm in analogous fashion to the response to disruption of *Lsg1* in *Saccharomyces cerevisiae* (Hedges et al., 2005). However, rather than causing accumulation of pre‐60S subunits and decreased polysomes as in *S. cerevisiae*, it resulted in an increase in translation accompanied by normal ribosome content, consistent with previous reports of senescence as a highly metabolically active process requiring elevated rates of translation (Dörr et al., 2013; Narita et al., 2011). Similarly, deletion of *Sbds* in normal mouse pancreas was recently shown to elicit a senescent response without perturbation of global ribosome content (Tourlakis et al., 2015), whilst the equivalent perturbation in *S. cerevisiae* promotes impairment of the polysome profile (Menne et al., 2007). Taken together, these reports suggest that an important function of the senescence response may be to halt cellular proliferation prior to the onset of a translational defect, thereby protecting cellular translational capacity in response to perturbations of ribosome maturation. *S. cerevisiae* lacks the ability to mount complex stress responses such as senescence, and therefore, these 60S defects result in catastrophic reduction of ribosome content.

Senescence is a pleiotropic response to many cellular stresses, and although many of the effector pathways (e.g., cell cycle arrest and the SASP) are well characterized, the precise molecular mechanisms that trigger senescence remain obscure in most cases. Transcriptomic analyses can shed light upon molecular mechanisms of cellular stresses because discrete effector pathways often reveal the nature of the initial stress, for example the induction of NRF2 gene targets in response to oxidative stresses (reviewed in Nguyen, Nioi, and Pickett (2009)) and HIF1 gene targets upon hypoxia (reviewed in Kaluz, Kaluzová, and Stanbridge (2008)). Our transcriptomic analyses gave a clear indication of stress arising at the ER, and our further analyses revealed disruption of ER morphology upon loss of LSG1. The origin of a cellular stress response at the ER is consistent with previous reports of LSG1 and RPL10 localization and function at the ER (Loftus et al., 1997; Reynaud et al., 2005). It is unlikely that the LSG1‐/RPL10‐mediated removal of NMD3 only occurs at the ER and we favour a model whereby this reaction occurs throughout the cytosol and at the ER, but the stress response arises due to perturbation of the latter. At this time, it is unclear why there is such a specific activation of the cholesterol biosynthesis pathway by shLSG1 and why this signature is also so prevalent in OIS.

A recent study identified accumulation of the ribosomal 40S subunit protein RPS14 as a mechanism contributing to senescence in response to multiple stimuli (Lessard et al., 2018). Unlike our p53‐dependent response, the response to RPS14 was Rb‐dependent, indicating that it is mechanistically distinct. Although the p53 pathway is required for the induction of senescence upon knockdown of LSG1 in primary human fibroblasts, we observed a strong senescence response in cells transformed with Ras/E6E7, despite the absence of p53 activity. Ras‐induced senescence is associated with enhanced intracellular metabolic activity through several pathways (Dörr et al., 2013; Herranz et al., 2015; Laberge et al., 2015; Narita et al., 2011), and whilst E6E7 relieves the proliferative block, it enhances the SASP and may elevate levels of metabolic stresses. We suggest that knockdown of LSG1 in such cells may compound these stresses, leading to the induction of a p53‐independent senescence response. Indeed, the cholesterol biosynthesis signature is induced by Ras and then further enhanced by knockdown of LSG1, perhaps reflecting enhanced metabolic stress arising at the ER. Overall, an emerging picture is that cells use multiple mechanisms to surveil ribosome biogenesis and that senescence is the outcome when defects are detected.

The inhibition of 60S ribosomal subunit maturation gives rise to a robust senescence response that is comparable with the OIS induced by oncogenic Ras in all aspects that we examined except for the SASP. The SASP elicited by deregulation of Ras in fibroblasts is a cocktail of pro‐inflammatory cytokines and chemokines resembling those produced during an immune response to infection. On the other hand, the restricted SASP activated upon inhibition of 60S maturation primarily involves components of the TGFβ signalling pathway. Furthermore, in Ras/E6E7 cells, where the SASP is elevated, knockdown of LSG1 reduced the expression of markers of the SASP. In terms of a potential cancer therapy, inhibition of the strongly pro‐inflammatory SASP is likely to be a considerable advantage, since pro‐inflammatory signalling, through IL‐6 in particular, has been linked to tumour progression and metastasis (He et al., 2013; Kim et al., 2009).

A therapeutic concept that is supported by our data is that inhibition of ribosome biogenesis could be an effective cancer therapy and our induction of tumour suppression in transformed cells with defective p53 and RB pathways is particularly encouraging. GTPases have not previously been strong candidates for inhibition through small molecules, although the translational GTPase eEF2, a homologue of EFL1, has been well validated as an inhibitory target since the naturally occurring inhibitors sordarin, diphtheria toxin and exotoxin A all target this enzyme. Inhibition of eEF2 is toxic to mammalian cells due to inhibition of translation, but here, we demonstrate that inhibition of LSG1, and possibly EFL1 by extension, may provide an effective prosenescent cancer therapy with lesser side effects since translation remains unimpaired. Recently, an important advance in the field of GTPase inhibition was reported with the identification of a non‐nucleotide active site inhibitor of the small GTPase Rab7 that can act as a scaffold for derivatization to produce inhibitors of other GTPases (Agola et al., 2012; Hong et al., 2015). Accordingly, the translational GTPases of 60S ribosomal subunit maturation may be amenable to development of inhibitors. In conclusion, this study suggests that the GTPase LSG1 has high potential as a candidate target for prosenescent cancer therapy in cases where tumour‐suppressive senescence is bypassed due to p53 and/or RB deficiency.

## 4 METHODS

### 4.1 Cell culture

MRC5 and IMR90 early passage primary human fibroblasts were purchased from the Culture Collection at Public Health England. These cells and HEK293ET (used for viral production—a kind gift of Felix Randow at the MRC Laboratory of Molecular Biology, Cambridge) were cultured in Dulbecco's Modified Eagle's Medium (DMEM—Thermo Fisher, 41965) supplemented with 10% foetal bovine serum (FBS—Thermo Fisher, 10270–106) and 1% penicillin/streptomycin. All cells were maintained between 20% and 90% confluence at 37°C, 5% CO_2_.

### 4.2 shRNA design and cloning

The Whitehead Institute for Biomedical Research small interfering RNA (siRNA) design tool (http://jura.wi.mit.edu/bioc/siRNAext/) was used to opt for the top‐scoring 21‐mer target sequences for our genes of interest. These oligos were run through NCBI's BLAST program to minimize off‐target effects. shRNA design was conducted in compatibility with the lentiviral transfer vector pLKO.1. Forward and reverse oligos (purchased from Sigma) were annealed and ligated into the viral transfer vector and then used to transform competent bacteria cells (protocol at: http://www.addgene.org/tools/protocols/plko/). Successful clones were identified by restriction digestion and sequencing.

### 4.3 Viral transduction

Lentiviral production for shRNAs was carried out by transfection of HEK293ET cells with a packaging vector (psPAX2), VSV‐G envelope (pMD2.G) and viral transfer vector (listed below). For retroviral transduction, pGag‐Pol was used in place of psPAX2. 10 μg of each vector was combined with 80 μl of polyethyleneimine (PEI—1 μg/μl) in a 500 μl volume (the remainder being DMEM). This was then added to a 75‐cm^2^ flask of cells containing 10 ml of DMEM/10% FCS and incubated overnight. Medium was exchanged the following day for DMEM/10% FCS and left for a further 24 hr at which point the viral supernatant was harvested for infection. Viral supernatant was diluted (typically 3:10) with DMEM/10% FCS and mixed with polybrene (hexadimethrine bromide) at a final concentration of 5 μg/ml. This was filtered through a sterile 0.45‐μm filter and used to replace the medium on recipient cells. Twenty‐four hours after infection, the virus‐containing medium was replaced with fresh medium. Forty‐eight hours after infection, antibiotic was added for selection, puromycin at 1 μg/ml or blasticidin at 5 μg/ml, and cells were selected until uninfected control cells had died. Time points referred to are days postinfection (not selection).

### 4.4 Viral transfer vectors

Knockdown of 60S maturation factors using lentivirus was carried out using pLKO1 or TetLKO‐puro containing oligos as follows:

Ctrl: CCGGTCCGCAGGTATGCACGCGTG
LSG1: CCGGTGGGCTACCCTAATGTTGGTACTCGAGTACCAACATTAGGGTAGCCCATTTTTG
SBDS(a): CCGGAAGCTTGGATGATGTTCCTGACTCGAGTCAGGAACATCATCCAAGCTTTTTTTG
SBDS(b): CCGGCTGCTTCCGAGAAATTGATGACTCGAGTCATCAATTTCTCGGAAGCAGTTTTTG

E6, E7 and E6E7 constructs in pLXSN have been previously described (Acosta et al., 2008). Dominant‐negative p53 (GenBank KF766124) and KRas^G12V^ were expressed in the retroviral vector pM6P‐Blast (a kind gift of Felix Randow, MRC Laboratory of Molecular Biology).

### 4.5 siRNA transfections

For siRNA transfections, plated cells were treated with a mix of medium containing the siRNA SMARTpool or single siRNA at a final concentration of 50 nM and 3.5% Hiperfect transfection reagent (Qiagen, 1029975). For long‐term silencing, siRNA transfections were repeated every 3 days. The siRNA sequences are listed below:

**Table:** 

siRNA	Sequence
NTC	UGGUUUACAUGUCGACUAA
	UGGUUUACAUGUUGUGUGA
	UGGUUUACAUGUUUUCUGA
	UGGUUUACAUGUUUUCCUA
siEFL1	ACAUGAAGCAUGUCGCUAU
	ACAUGAACGCAGUACGAAA
	AAAGAGAGAAGGUCGGGUA
	GCCAGUAGAUACCGAGAUU
siLSG1	GAAAUGACUUGCAGCGGAA (1)
	AGAUAGUAGAUGCUCGAAA (2)
	AGGGAUGGUUCACGAGACA (3)
	GCCAAUAAGGAGAACGUCA (4)

### 4.6 Western blotting and antibodies

Cells were lysed in cell lysis buffer (Cell Signaling, 9803S) supplemented with EDTA‐free protease inhibitors for 10 min on ice. Protein content quantification and normalization were performed by Bradford assay. Lysates were separated by sodium dodecyl sulphate (SDS)–polyacrylamide gel electrophoresis (PAGE) and transferred to polyvinylidene difluoride (PVDF) membrane in a wet‐transfer manner. The membrane was incubated in blocking buffer (5% nonfat milk/Tris‐buffered saline (TBS)/0.1% Tween‐20) for 1 hr at room temperature (RT) for the blocking of nonspecific sites. Primary and HRP‐linked secondary antibodies were diluted in blocking buffer. Primary antibody incubation was o/n, whereas secondary antibody lasted for 1 hr at RT. The SuperSignal West Pico Chemiluminescent Substrate (Thermo Scientific, 34079) was used for signal development, which was digitally detected in a Biorad detector (731BR00785) and analysed using the software Image lab 4.1. Antibodies used were raised against: LSG1 (Proteintech 17750), EFL1 (24729), SBDS (Abcam ab128946), RPL28 (Proteintech 16649), BrdU (Pharmingen 558599), p53 (Santa Cruz sc‐126), p16 (Santa Cruz sc‐56330), p21 (Sigma p1484), pST/Q (Cell Signaling 2851), IL‐1α (R&D MAB200), IL‐1β (R&D MAB201), IL‐6 (R&D AF206NA), IL‐8 (R&D MAB208), Ki67 (Invitrogen 180191Z), calnexin Alexa Fluor 647 conjugate (Abcam ab202572), SQLE (Bethyl Laboratories A304‐590A‐T) and HMGCS1 (Proteintech 12544‐1‐AP).

### 4.7 Immunofluorescence staining and High Content Microscopy

High content microscopy was performed as previously described (Hari & Acosta, 2017). Where included, cells were treated with 50 mM BrdU (Sigma, 858811) for 16 hr prior to fixation. Briefly, cells were fixed with 10% formalin for 10 min, permeabilized with 0.2% Triton/PBS for 10 min and then blocked with blocking solution (1% BSA/0.2% fish gelatin in PBS) for 1 hr. Primary antibody diluted in blocking solution was then added, and the cells were incubated for 1 hr at RT. Anti‐BrdU solution was supplemented with 0.5 U/μl DNAse (Sigma D4527) and 1 mM MgCl_2_. Incubation with fluorescent secondary antibodies for 1 hr and 1 μg/ml DAPI for 30 min followed. The cells were visualized using confocal microscopy. 96‐well plates were scanned using an ImageXpress Micro High Content Imaging System (Molecular Devices), acquiring multiple images. Automated analysis of these images was performed using the software MetaXpress 5.1.0.46 (Molecular Devices).

### 4.8 Cytochemical staining for SA‐β‐galactosidase

Cell fixation was performed in 0.5% glutaraldehyde/PBS for 15 min at RT. After washes in 1 mM MgCl_2_/PBS, pH 6, the cells were incubated in staining solution [2 mg/ml 5‐bromo‐4‐chloro‐3‐indolyl‐β‐D‐galactopyranoside (Sigma, B4252), 1.64 mg/ml K_3_Fe(CN)_6_, 2.1 mg/ml K_4_Fe(CN)_6_.3H_2_O in 1 mM MgCl_2_ 1, pH 6] at 37°C, for 24 hr. The production of a blue precipitate within the cytoplasm, as observed under an inverted microscope, determined the lysosomal SA‐β‐gal activity (Dimri et al., 1995).

### 4.9 O‐propargyl puromycin assay

For the OPP assay, the Click‐iT Plus OPP Alexa Fluor 488 kit (Thermo Fisher, C10456) was used, following the manufacturer's protocol. Briefly, transduced MRC5 cells were labelled with 20 μM OPP in dimethyl sulfoxide (DMSO) for 30 min. O‐propargyl puromycin intensity detection was performed by standard immunofluorescence procedures, followed by high content microscopy.

### 4.10 Cell apoptosis assay

The cell apoptosis assay was carried out using the Annexin V and Dead Cell Assay Kit on the Muse (Merck Millipore) according to manufacturer's instructions.

### 4.11 Quantitative real‐time PCR

RNA was extracted using the RNeasy Mini Kit (Qiagen, 74104) and reverse‐transcribed with the QScript enzyme (Quanta, 95048), according to the manufacturer's protocol. The cDNA obtained was used as a template for qPCR. Primers were used at 200 nM each in a total reaction volume of 20 μl. SYBR Select Master Mix (Applied Biosystems, 4472908) was used for the reaction, which was performed in a StepOne Real‐Time PCR system (Applied Biosciences), using the software StepOne v2.3. The primer oligos are listed below:

Actin: CATGTACGTTGCTATCCAGGC / CTCCTTAATGTCACGCACGAT
SerpinE1: CCTGGCCTCAGACTTCGGGGT / GGGGCCATGCCCTTGTCATCAAT
TGFB2: TGATCCTGCATCTGGTCACG / ATGGCATCAAGGTACCCACA
18S: GATGGTAGTCGCCGTGCC / GCCTGCTGCCTTCCTTGG
28S: AGAGGTAAACGGGTGGGGTC / GGGGTCGGGAGGAACGG
MSMO1: ATGCTTTGGTTGTGCAGTCA / TCACACAAAAGCACGATTCC
DHCR7: GACAACTGGATCCCACTGCT / TCCGAGGGTTAAACTCGATG
MVD: GTGTCTACGGCGTGGAGAGT / ACGGTACTGCCTGTCAGCTT
HMGCS1: TCTAGCTCGGATGTTGCTGA / AACAGATGCAAGGGAACCAT
SQLE: GTCTCCGGAAAGCAGCTATG / AAAAGCCCATCTGCAACAAC
FDFT1: ATAACCAATGCACTGCACCA / CCTTTCCGAATCTTCACTGC
IL1A: AGTGCTGCTGAAGGAGATGCCTGA / CCCCTGCCAAGCACACCCAGTA
IL1B: TGCACGCTCCGGGACTCACA / CATGGAGAACACCACTTGTTGCTCC
IL8: GAGTGGACCACACTGCGCCA / TCCACAACCCTCTGCACCCAGT
p21: CCTGTCACTGTCTTGTACCCT / GCGTTTGGAGTGGTAGAAATCT
p16: CGGTCGGAGGCCGATCCAG / GCGCCGTGGAGCAGCAGCAGCT
p53: CCGCAGTCAGATCCTAGCG / AATCATCCATTGCTTGGGACG
LSG1: ACTTTCAGACTCTCTATGTGG / AAACTAGTGATACAGGAGGAAC

### 4.12 Transcriptomic analysis

RNA was harvested from cells using an RNeasy/QIAshredder (Qiagen) protocol following the manufacturer's instructions. Reverse transcription of DNA was carried out using QScript enzyme (Quanta), and RNA was submitted to the Genome analysis core at the Wellcome Trust Clinical Research Facility (Western General Hospital) for AmpliSeq library preparation and IonTorrent sequencing. RNA samples were assessed for quality on the Agilent Bioanalyser with the RNA Nano chip, providing an RNA Integrity Number (RIN). Samples were quantified using the Qubit® 2.0 fluorometer and the Qubit® RNA Broad Range assay. 10 ng of RNA was reverse‐transcribed to make cDNA, and then, target genes were amplified for 12 cycles of PCR using the Ion AmpliSeq™ Human Gene Expression Core Panel, which contains a pool of 20,802 amplicons (41,604 primers) of approximately 150 bases in length. Ion Torrent sequencing adapters and barcodes were ligated to the amplicons, and adapter‐ligated libraries were purified using AMPure XP beads. Libraries were quantified by qPCR and diluted to 100 pM. Templates were prepared using the Ion PI Hi‐Q OT2 200 Kit and sequenced using the Ion PI Hi‐Q Sequencing 200 Kit. The Ion Proton platform was used to process the sequencing. Analysis of the data was performed using the Babelomics‐5 application ( http://babelomics.bioinfo.cipf.es ). The sample replicates were normalized using trimmed mean of M values (TMM) method and subjected to the Benjamini and Hochberg false discovery rate multiple test correction method to adjust the *p*‐value. AmpliSeq transcriptomic data have been deposited at the Gene Expression Omnibus under the accession number GSE128055. Hierarchical clustering analysis was performed using Cluster 3 software (Stanford University), and visualization was performed using TreeView 3.0 software (Princeton University). Preranked gene lists by fold change were subjected to gene set enrichment analysis (GSEA) using the GSEA 3.0 software from the Broad Institute (www.gsea msigdb.org ). Preranked gene expression transcriptomes were interrogated against gene set data bases at the Broad Institute repository. The enrichment statistics used was adjusted to weighted. The maximum and minimum size of the sets was adjusted to 500 to 10, respectively. The number of permutations was adjusted to 1,000. The normalization mode was meandiv. The Gene Ontology analysis of gene lists was performed using the DAVID functional annotation tool (https://david.ncifcrf.gov/tools.jsp).

### 4.13 Polysome profiles

Cells were lysed in detergent lysis buffer A (10 mM Tris‐HCl at pH 7.4, 10 mM NaCl, 1.5 mM MgCl_2_, 0.5% [v/v] Triton X‐100, 0.5% [w/v] deoxycholate, 1% [v/v] Tween‐20, 100 μg/ml cycloheximide) with complete EDTA‐free protease inhibitors (Roche) and 0.5 U/ml RNase inhibitor (Promega) and incubated for 10 min on ice. Lysates were cleared in a microfuge. Equal amounts (typically 10–20 A254 U) were applied to a 10%–50% (w/v) sucrose gradient in 11 ml of buffer B (10 mM Tris‐HCl at pH 7.4, 75 mM KCl, 1.5 mM MgCl_2_) and centrifuged (Beckman SW41 rotor) at 207,570 *g* for 80 min at 4°C. Samples were unloaded using a Brandel gradient fractionator, the polysome profiles were detected using a UV monitor (Gilson) at A254, and fractions were collected. For analysis of polysome‐associated mRNAs, polysomal fractions were pooled and RNA was purified using the RNeasy Mini Kit (Qiagen). RNA was reverse‐transcribed with QScript enzyme (Quanta) according to the manufacturer's protocol, and the cDNA was used as a template for PCR. Primer oligos used were as follows:

p16: CGGTCGGAGGCCGATCCAG / GCGCCGTGGAGCAGCAGCAGCT
p21: CCTGTCACTGTCTTGTACCCT / GCGTTTGGAGTGGTAGAAATCT
IL1α: AGTGCTGCTGAAGGAGATGCCTGA / CCCCTGCCAAGCACACCCAGTA
β‐actin: CATGTACGTTGCTATCCAGGC / CTCCTTAATGTCACGCACGAT

### 4.14 Crystal violet assay

Cells were seeded at low density and cultured for 15 days. Fixation was performed in 1% glutaraldehyde/PBS for 1 hr, at RT. After two H_2_O washes, the plates were left to dry for 1 day. Staining was performed using 0.15% crystal violet/H_2_O for 2 hr at room temperature, followed by washes with tap water. The dishes were dried, and photographs were taken using a document scanner. For relative quantitation purposes, the crystal violet was extracted in 1 M acetic acid/H_2_O during an overnight incubation and the absorbance at 595 nm was measured.

### 4.15 Statistics

Experiments were performed with biological triplicates and repeated multiple times. Error bars are standard deviations. *p*‐Values are derived from two‐tailed *t* tests or one‐way analysis of variance (ANOVA) with the appropriate post hoc correction for multiple comparisons as indicated in the figure legends. Significance symbols are as follows: *p < 0.05; **p < 0.01; ***p < 0.001.

## CONFLICT OF INTEREST

None declared.
