# Supplementary material for: Inhibition of the 60S ribosome biogenesis GTPase LSG1 causes endoplasmic reticular disruption and cellular senescence
Source: Aging Cell. 2019 May 31;18(4):e12981. doi: 10.1111/acel.12981 (PMC6612703; doi:10.1111/acel.12981)
Supplement: Supplementary file 1 [file ACEL-18-e12981-s001.pdf]

a.

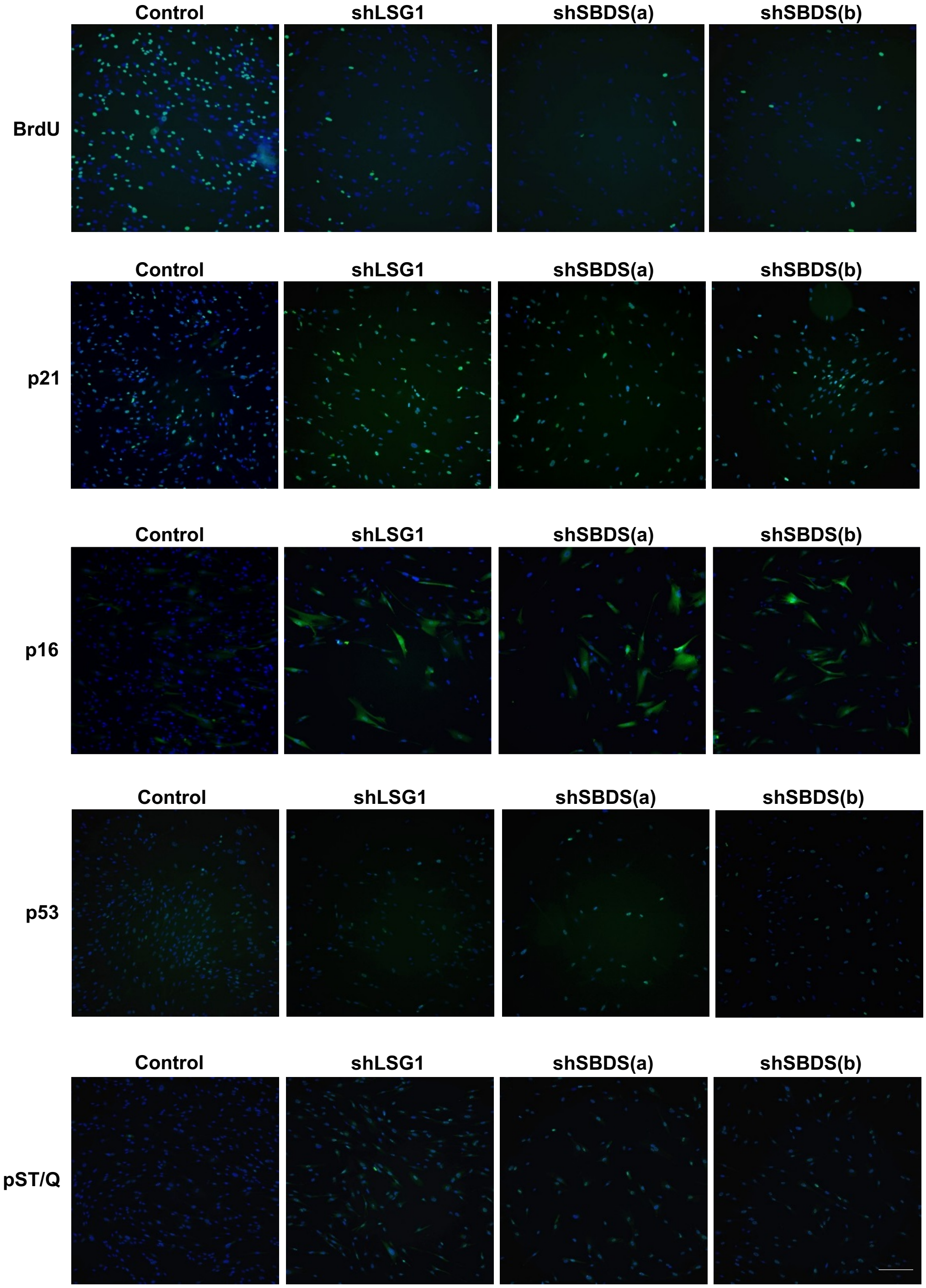
Control
shLSG1
shSBDS(a)
shSBDS(b)
BrdU
p21
p16
p53
pST/Q

b.

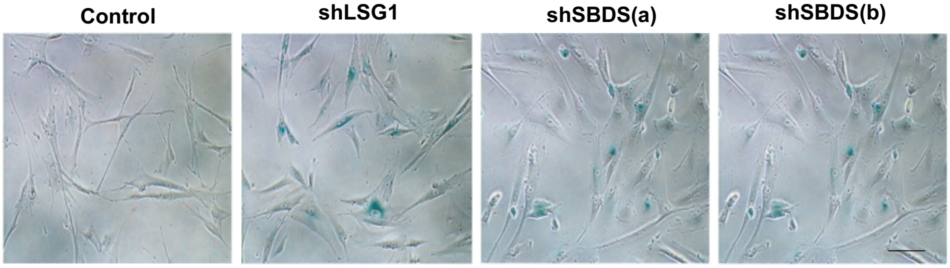
Control
shLSG1
shSBDS(a)
shSBDS(b)

Supplementary Figure 2. Pantazi *et al.*

a.

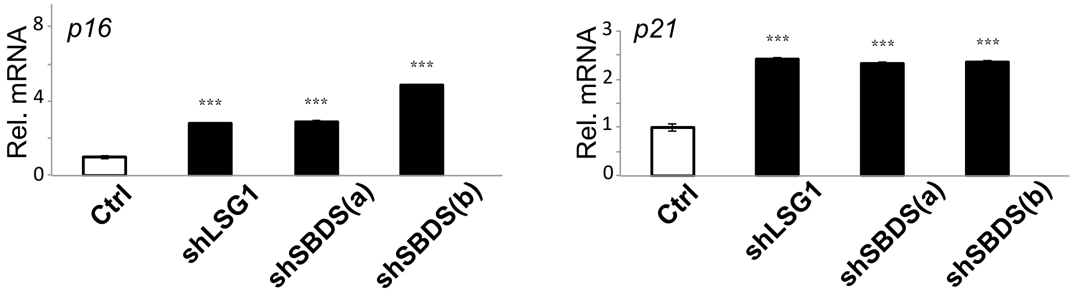

b.

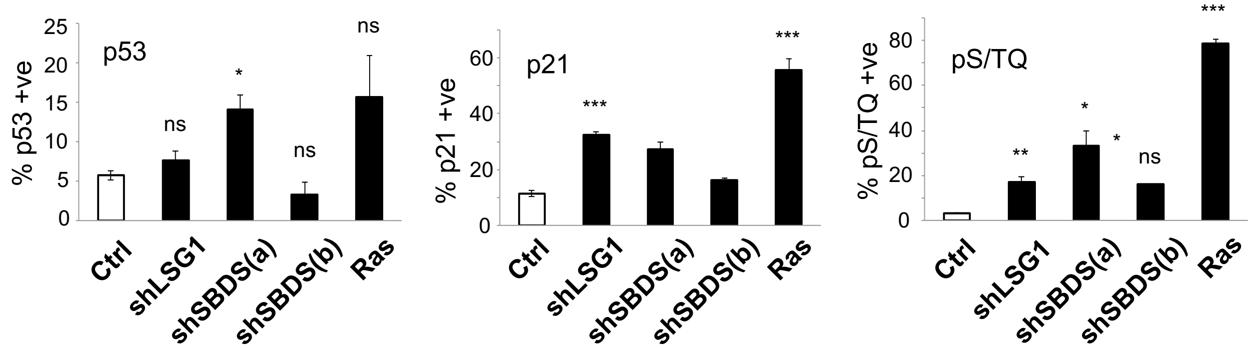

c.

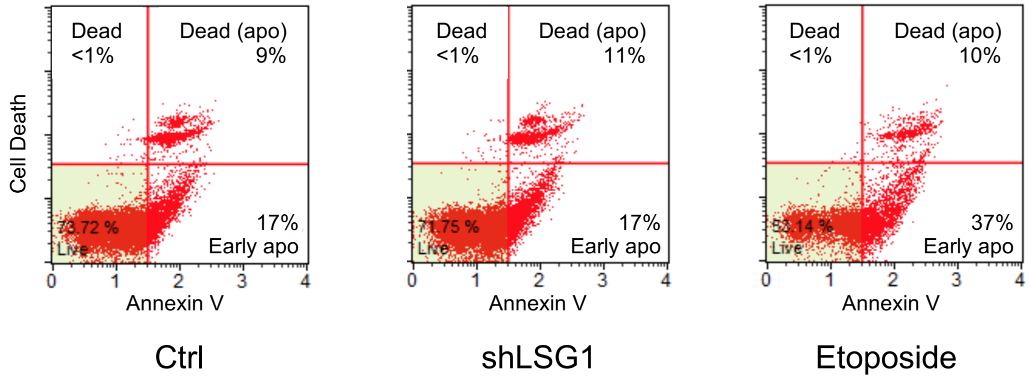

d.

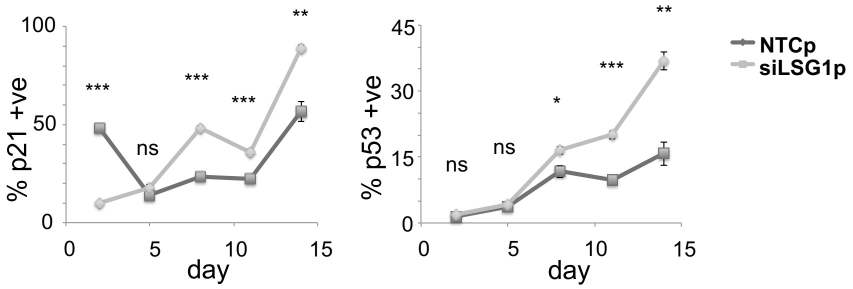

a.

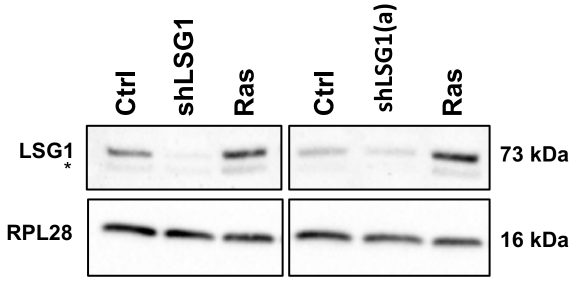
Ctrl
shLSG1
Ras
Ctrl
shLSG1(a)
Ras
LSG1
*
RPL28
73 kDa
16 kDa

b.

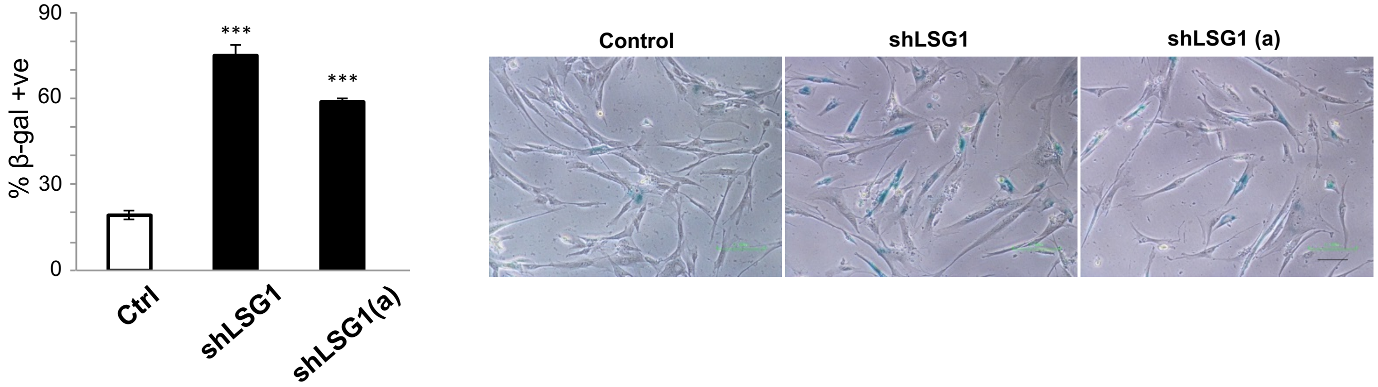
% β-gal +ve
90
60
30
0
***
***
Ctrl
shLSG1
shLSG1(a)
Control
shLSG1
shLSG1 (a)

c.

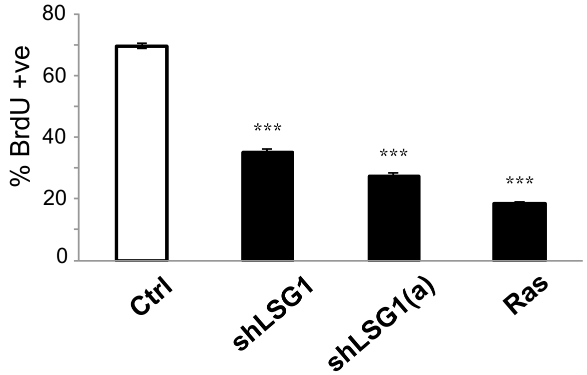
% BrdU +ve
80
60
40
20
0
***
***
***
Ctrl
shLSG1
shLSG1(a)
Ras

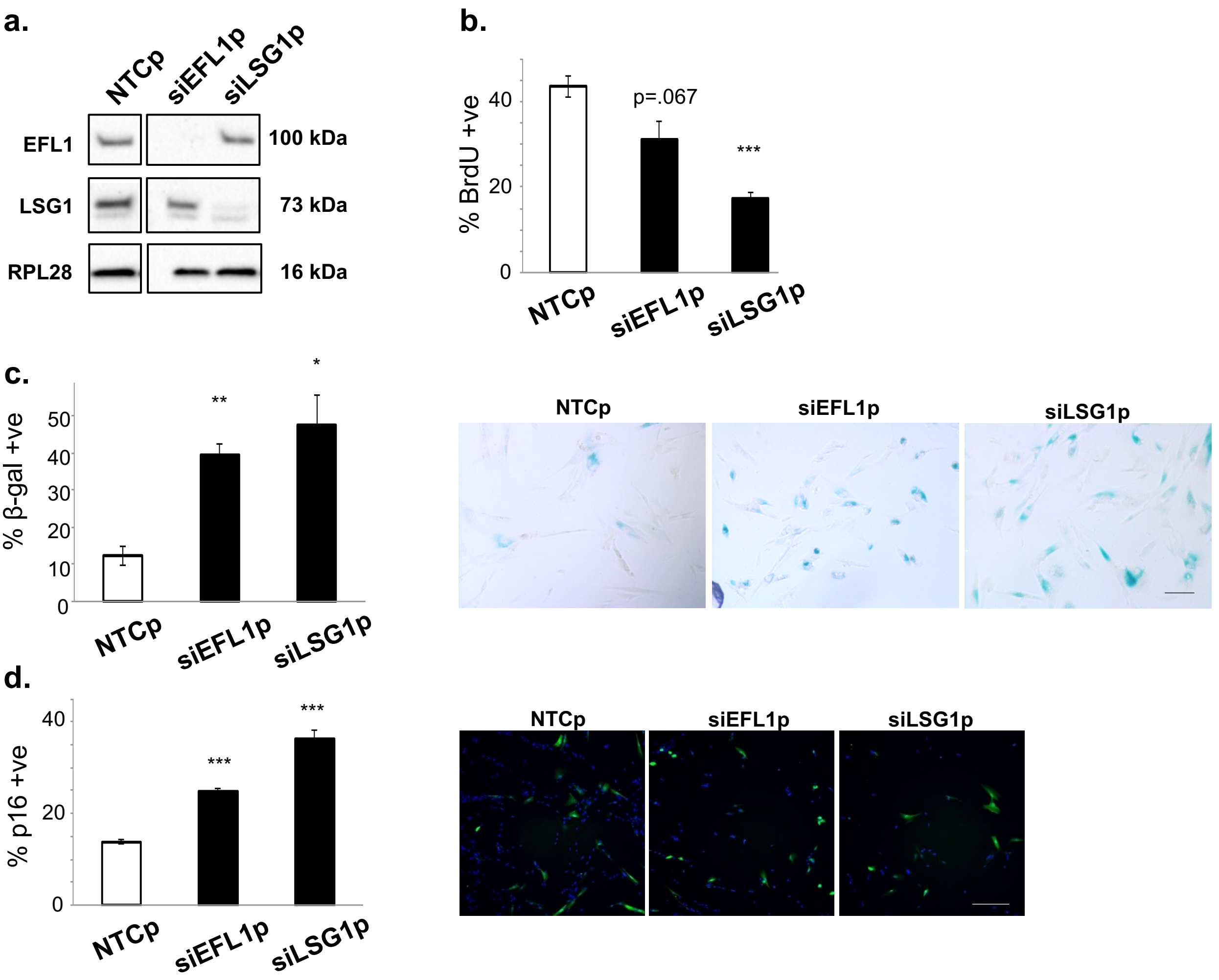
a.
NTCp
siEFL1p
siLSG1p
EFL1
100 kDa
LSG1
73 kDa
RPL28
16 kDa
b.
% BrdU +ve
40
20
0
p=.067
***
NTCp
siEFL1p
siLSG1p
c.
% β-gal +ve
50
40
30
20
10
0
**
*
NTCp
siEFL1p
siLSG1p
NTCp
siEFL1p
siLSG1p
d.
% p16 +ve
40
20
0
***
***
NTCp
siEFL1p
siLSG1p
NTCp
siEFL1p
siLSG1p

a.

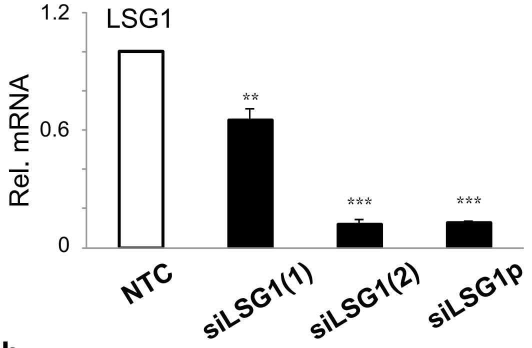
LSG1
Rel. mRNA
1.2
0.6
0
**
***
***
NTC
siLSG1(1)
siLSG1(2)
siLSG1p

b.

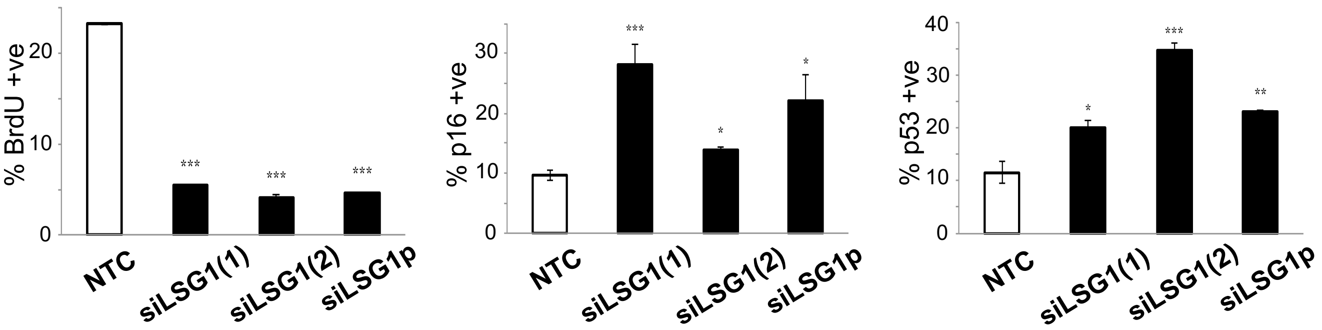
% BrdU +ve
20
10
0
***
***
***
NTC
siLSG1(1)
siLSG1(2)
siLSG1p
% p16 +ve
30
20
10
0
***
*
*
NTC
siLSG1(1)
siLSG1(2)
siLSG1p
% p53 +ve
40
30
20
10
0
*
***
**
NTC
siLSG1(1)
siLSG1(2)
siLSG1p

a.

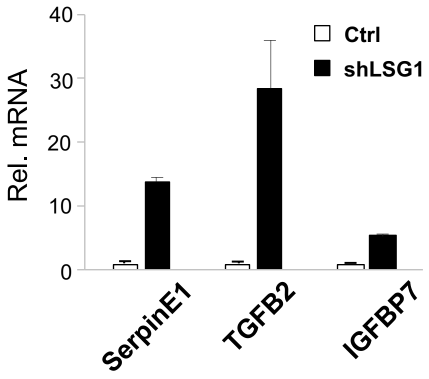

b.

List of cholesterol biosynthesis-related genes enriched in shLSG1-induced senescence

**Cholesterol biosynthesis**			
**Gene**	**Name**	**Fold-change**	**Position in list**
MSMO1	Methylsterol monooxygenase 1	14.16	1
HMGCS1	3-Hydroxy-3-Methylglutaryl-Coenzyme A Synthase 1	14.13	3
CYP51A1	Cytochrome P450 Family 51 Subfamily A Member 1	10.41	4
ANGPTL4	Angiopoietin Like 4	10.09	
TRIB1	Tribbles Pseudokinase 1	9.62	8
IDI1	Isopentenyl-Diphosphate Delta Isomerase 1	9.34	
DHCR7	7-Dehydrocholesterol Reductase	9.27	
SQLE	Squalene Epoxidase	9.08	
HMGCR	3-Hydroxy-3-Methylglutaryl-CoA Reductase	8.91	
FDFT1	Farnesyl-Diphosphate Farnesyltransferase 1	8.89	
MVK	Mevalonate Kinase	6.42	
LIPA	Lipase A	6.38	
MVD	Mevalonate Diphosphate Decarboxylase	6.15	
HSD17B7	Hydroxysteroid (17-beta) dehydrogenase 7	3.52	
NSDHL	NAD(P) Dependent Steroid Dehydrogenase-Like	3.31	

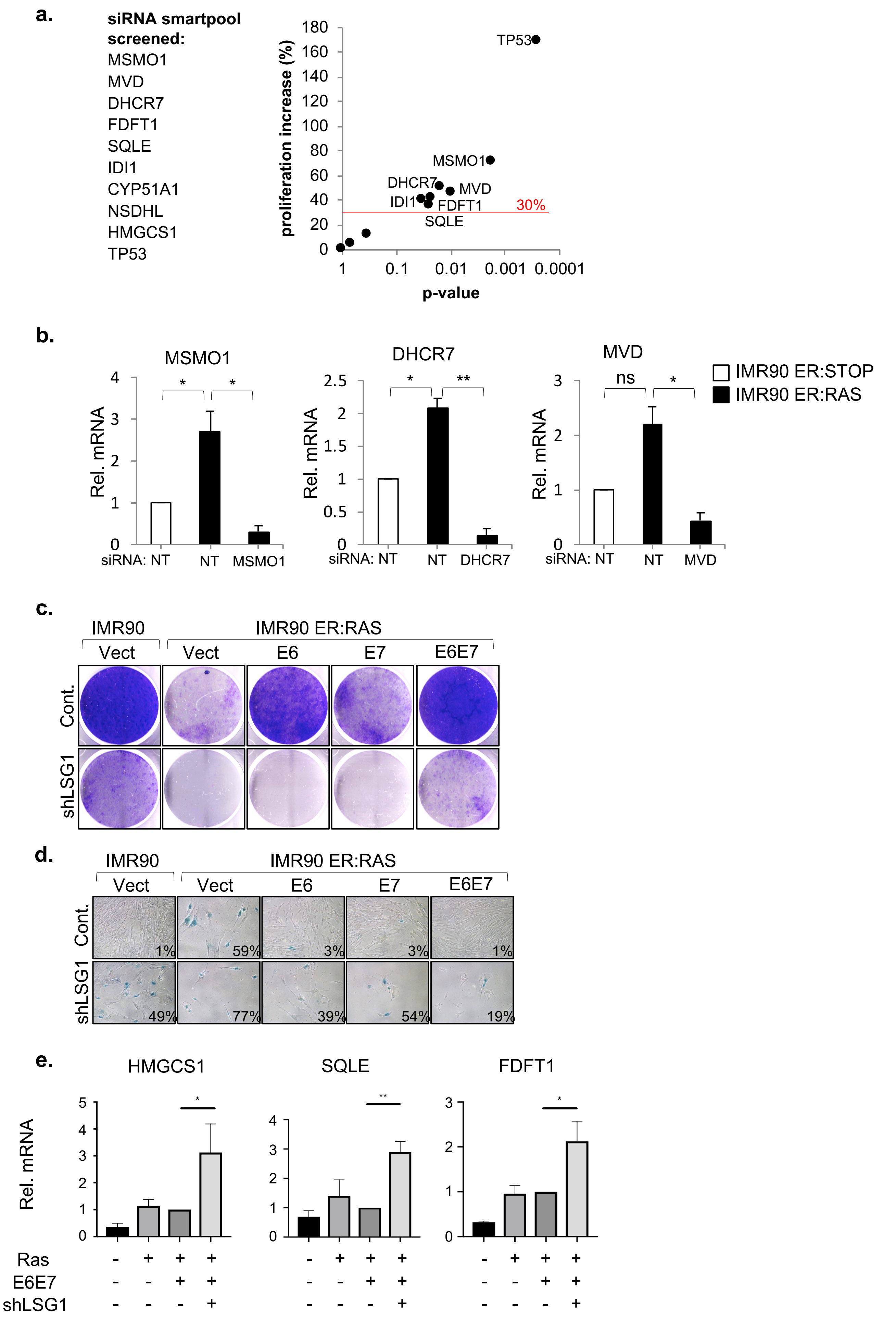
a.
siRNA smartpool screened:
MSMO1
MVD
DHCR7
FDFT1
SQLE
IDI1
CYP51A1
NSDHL
HMGCS1
TP53
proliferation increase (%)
p-value
TP53
MSMO1
DHCR7
MVD
IDI1
FDFT1
SQLE
30%
b.
MSMO1
DHCR7
MVD
Rel. mRNA
siRNA: NT
NT
MSMO1
DHCR7
MVD
ns
IMR90 ER:STOP
IMR90 ER:RAS
c.
IMR90
IMR90 ER:RAS
Vect
E6
E7
E6E7
Cont.
shLSG1
d.
IMR90
IMR90 ER:RAS
Vect
E6
E7
E6E7
Cont.
shLSG1
1%
59%
3%
3%
1%
49%
77%
39%
54%
19%
e.
HMGCS1
SQLE
FDFT1
Rel. mRNA
Ras
E6E7
shLSG1
